# Ginsenoside Rb1 Improves Cognitive Impairment Induced by Insulin Resistance through Cdk5/p35-NMDAR-IDE Pathway

**DOI:** 10.1155/2020/3905719

**Published:** 2020-05-20

**Authors:** Ranyao Yang, Xue Jiang, Xiqian He, Donglou Liang, Shusen Sun, Guangyan Zhou

**Affiliations:** ^1^Department of Clinical Pharmacy, Jining No. 1 People's Hospital, Jining Medical University, Jining, Shandong, China; ^2^The State Key Laboratory of Pharmaceutical Biotechnology, The University of Hong Kong, Hong Kong, China; ^3^Department of Medicine, The University of Hong Kong, Hong Kong, China; ^4^Joint Laboratory of Guangdong and Hong Kong on Metabolic Diseases, Guangdong Pharmaceutical University, Guangzhou, Guangdong, China; ^5^Department of Rehabilitation Medicine, Jining No. 1 People's Hospital, Jining Medical University, Jining, Shandong, China; ^6^Department of Pharmacy Practice, Western New England University, Springfield, USA; ^7^Department of Emergency, Jining No. 1 People's Hospital, Jining Medical University, Jining, Shandong, China

## Abstract

The relationship between diabetes mellitus (DM) and Alzheimer's disease (AD) has attracted wide attention. Studies have reported that ginsenoside Rb1 can improve human cognitive ability and glucose tolerance during the development of diabetes. The mechanism behind the improvement in cognitive ability and glucose tolerance still remains unclear. In this study, streptozotocin- (STZ-) injected mice were used as models to explore the mechanisms behind the cognitive improvement of ginsenoside Rb1. According to the results of behavioral tests, ginsenoside Rb1 improved memory and cognitive ability of STZ-lesioned mice. In addition to that, ginsenoside Rb1 also relieved glucose intolerance induced by STZ injection by enhancing insulin sensitivity. These beneficial effects of ginsenoside Rb1 is most likely mediated by upregulating the expression of NMDAR1 and IDE in the hippocampus through inhibiting the activity of Cdk5/p35. This work will be of great importance in illustrating the mechanisms of ginsenoside Rb1 for improving cognitive ability, as well as revealing the relationship between diabetes and AD.

## 1. Introduction

Alzheimer's disease (AD) is a complex neurodegenerative disorder with insidious onset and slow progression. AD is the 5^th^ leading cause of death in elderly individuals [1], which has been paralleled by the increase of complications from coexisting diabetes mellitus (DM). Currently, over 400 million people suffer from DM in the world, and this number is predicted to double by 2030 [2]. Increasing clinical and basic studies suggest that DM is strongly associated with cognitive dysfunction, with many brain structures being sensitive to changes in brain insulin resistance and deficiency [3]. Both AD and DM are connected with impaired insulin signalling, amyloid beta (A*β*) formation, neurofibrillary tangle formation, neuroinflammation, glycogen synthase kinase 3*β* (GSK3*β*) signalling, neuronal apoptosis, acetylcholine esterase activity regulation, and oxidative stress injury [4–7]. Due to similar molecular and cellular mechanisms among type 1 diabetes (T1DM), type 2 diabetes (T2DM), and AD, AD has been referred to as ‘type 3 diabetes' by researchers [8, 9].

Ginseng is a traditional drug in China used to help enhance cognitive improvement. Ginseng has been used for AD and has been proven to have great efficacy in enhancing memory and improving cognitive function [10–13]. Ginsenoside Rb1 is a main bioactive ingredient in ginseng [14–15] and exerts significant positive effects in defending against oxidative stress and neuronal apoptosis, while enhancing spatial learning ability in A*β*-induced AD rat model [16–17]. Although studies using ginsenoside Rb1 on AD advanced our knowledge in revealing the pathology of AD, the possible mechanism involved still remains unclear. In addition, there are documents that support the benefits of ginsenoside Rb1 in antiobesity, antihyperglycaemic, and antidiabetic effects by regulating the metabolism of a glycolipid and improvement of insulin sensitivity [18–19], but the mechanism involved still remains largely unknown and awaits further exploration.

Due to ginsenoside Rb1 used as a cognitive and antidiabetic agent and the close relationship between AD and diabetes, we are very interested to explore the role of ginsenoside Rb1 in cognitive impairment induced by insulin resistance and the underlying mechanisms that lead to such benefits. Using STZ-injected mice as a cognitive impairment model, ginsenoside Rb1 was shown to shorten the latency into the platform and increase entry times across the target area in Morris water maze (MWM) test and prolong latency time and decrease error times of mice in the step-down test. In the hippocampus, ginsenoside Rb1 could stimulate the expression of NMDAR1 and IDE, which probably was mediated by the inhibition of the Cdk5/p35 activity. Our study, for the first time, provides a possible mechanism for the beneficial effects of ginsenoside Rb1 on cognitive ability in a diabetic mouse model, revealing the relationship between diabetes and AD.

## 2. Material and Methods

### 2.1. Drugs and Reagents

Ginsenoside Rb1 was purchased from Shanghai Pureone Biotechnology Company. Insulin was purchased from Novo Nordisk (Copenhagen, Denmark). Streptozotocin (STZ) was purchased from Sigma-Aldrich company. The primary antibody to N-methyl-daspartate receptor type 1 (NMDAR1) was purchased from Epitomics company. The primary antibody to insulin-degrading enzyme (IDE) was purchased from Abcam company. The horseradish peroxidase- (HRP-) conjugated secondary antibody and anti-*β*-actin were purchased from Millipore company. Cyclin-dependent kinase/p35 (Cdk5/p35) kinase assay for detection of enzyme activity was purchased from Promega company.

### 2.2. Animals and Treatments

All animal procedures in this study were conducted according to the regulations of the Institutional Animal Care and Use Committee of China on animal welfare and were approved by the Animal Care Committee of Jining Medical College. The C57BL/6N male mice were obtained from the Laboratory Animal Unit of Jining Medical College. Mice had free access to food and water, and they were maintained under standard animal housing conditions in a normal 12-hour light-dark cycle. When mice were housed for 15 weeks, they were treated with STZ that was dissolved in a sodium citrate buffer (pH = 4.5) via intraperitoneal administration at a dose of 150 mg/kg body weight [20] to induce a hyperglycaemic state. Age-matched control mice were injected with an isovolumetric vehicle. From then, the mice were randomly divided into 3 groups, including the control group (Veh+Veh), the model group (STZ+Veh), and the ginsenoside Rb1 group (STZ+30 mg/kg ginsenoside Rb1). There were 8-10 male mice in each group. Ginsenoside Rb1 was dissolved in distilled water. Mice received oral ginsenoside Rb1 or isovolumetric water every day until the mice were killed.

### 2.3. Morris Water Maze Test (MWM)

Four weeks after the treatment of ginsenoside Rb1 or vehicle, the MWM test was performed to assess the effects of ginsenoside Rb1 on mouse spatial learning and memory [21]. It consisted of a water tank (diameter, 120 cm; height, 40 cm) with four quadrants, with a hidden platform at a fixed position in one of the quadrants. The water temperature was 23.0 ± 1.0°C.

#### 2.3.1. Acquisition Phase

This part is also called the navigation trial. The mice were trained for five days, which consisted of two trials per day. Each mouse was released into a quadrant facing the wall of the water tank. Once the mice were in the water tank, they were given 60 seconds to reach the hidden platform. If the mouse failed to reach the platform, it would be guided to the platform, where it had to stay for 10 seconds. Latency (searching time) to platform and the number of entry times into the platform within 60 seconds were recorded by a camera hanging above the water tank [22].

#### 2.3.2. Probe Trial

After the 5-day training period, the platform was removed and an exploratory test was conducted to evaluate the spatial memory. In this trial, each mouse was regulated to swim for 60 seconds. The number of times the mice crossed the platform location was recorded [23–24].

### 2.4. Step-Down Test

A step-down test was conducted to evaluate memory in the mice. The mice underwent two trials, namely, training and test period over two days. During the training trial, each mouse was initially placed on the platform. If the mouse stepped down to the grid floor, an electric shock (36 V, 1.5 mA) would be delivered; then, it would jump back to the platform. A test trial was conducted 24 hours after the training trial. Duration (latency) on the platform and the number of times jumping off the platform (error times) were recorded within 300 seconds.

### 2.5. Glucose Tolerance Test (GTT)

The mice were fasted for 16 hours (18:00-10:00), with the fast followed by a 1.0 g/kg of a glucose solution diluted in saline that was injected intraperitoneally. Blood glucose levels were monitored from the tail at 0, 5, 15, 30, 45, 60, 75, 90, and 120 minutes postglucose injection with a OneTouch glucose meter (Roche, Accu-Chek Aviva Model).

### 2.6. Insulin Tolerance Test (ITT)

Mice were fasted for 6 hours (9:00-15:00), with the fast followed by 0.5 U/kg of insulin that was injected intraperitoneally. Blood glucose levels were monitored from the tail at 0, 20, 40, 60, 80, 100, and 120 minutes postinsulin injection.

### 2.7. Preparation of Mouse Brain Tissue

Mice were anesthetized using 2 mg of ketamine and 0.2 mg of xylazine via intraperitoneal injection. The brains of mice were separated, and the hippocampi were dissected and stored at -80°C until use was appropriate. The hippocampi were homogenized in a RIPA buffer supplemented with 0.2 mM PMSF (a protease inhibitor cocktail), then incubated for 20 minutes on ice and centrifuged for 10 minutes at 14,000×g and 4°C. The protein contents were detected using the bicinchoninic acid method (CoWin Bioscience Co., Beijing).

### 2.8. Cdk5/p35 Activity Measurement

Twenty micrograms of protein from the hippocampi was used to detect the activity of Cdk5/p35. ATP was involved in the kinase reaction of Cdk5/p35 phosphorylating its substrate, histone H1. The amount of ADP formed in this reaction could reflect the activity of Cdk5/p35. ADP was converted to ATP, which was converted into light by Ultra-Glo™ Luciferase. The luminescent signal positively correlated with the amount of ADP formed and Cdk5/p35 activity. In the kinase reaction, the final concentration of ATP, protein from hippocampi, and histone H1 were 3 *μ*M, 20 *μ*g, and 70 *μ*M, respectively. This mixture was incubated at 37°C for 60 minutes. The luminescent signals were detected using a luminescence microplate reader.

### 2.9. Western Blot Analysis

Twenty micrograms of protein from the hippocampi was separated by 10% SDS-PAGE before being transferred to a polyvinyldifluoride membrane (Millipore, MA, USA). After blocking with 2.5% BSA, the polyvinyldifluoride membranes were incubated with the primary antibody overnight at 4°C (anti-*β*-actin, 1 : 1000; anti-NMDAR1, 1 : 3000; and anti-IDE, 1 : 3000). Following the incubation with the primary antibody, membranes were incubated with the HRP-conjugated secondary antibody (1 : 3000) at room temperature for 1 hour. Protein expression was visualized by means of enhanced chemiluminescence and digitalized using ChemiDoc-It™ Imaging System (UVP, Upland, CA, USA). The gray value was analyzed using a Gel-Pro 32 (Media Cybernetics, Rockville, MD, USA).

### 2.10. Statistics

All statistical analyses were conducted using Prism 6 (GraphPad Software Inc., La Jolla, USA). Behavioral data from the training trial were analyzed using two-way ANOVA with repeated measures. Other statistical significance was determined by Student's *t*-test or one-way ANOVA. Data were reported as mean ± SEM, and *p* < 0.05 was considered to be significant.

## 3. Results

### 3.1. Ginsenoside Rb1 Inhibited the Activity of Cdk5/p35 *In Vitro*

**Figure 1:**
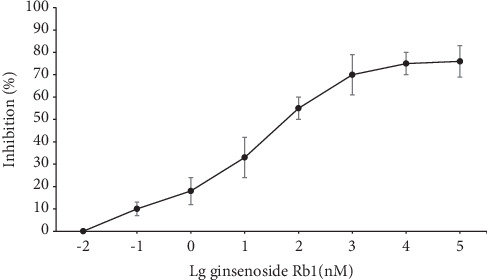
Ginsenoside Rb1 inhibited the activity of Cdk5/p35 (*n* = 6). Data are expressed as mean ± SEM.

Cdk5/p35 kinase assay was used for the detection of the enzyme activity. Ginsenoside Rb1 was dissolved in distilled water into the following concentrations: 0.01 nM, 0.1 nM, 1 nM, 10 nM, 100 nM, 1 *μ*M, 10 *μ*M, and 100 *μ*M. From then, the solution was incubated with the kinase reaction system (3 *μ*M ATP, 30 nM Cdk5/p35, and 70 *μ*M histone H1) at 37°C for 60 minutes. The luminescent signals were detected using a luminescence microplate reader. The results showed that ginsenoside Rb1 could inhibit the activity of Cdk5/p35 in a dose-dependent manner (Figure 1).

### 3.2. Ginsenoside Rb1 Improved Memory and Cognition of STZ-Lesioned Mice in Behavioral Tests

#### 3.2.1. Ginsenoside Rb1 Could Shorten the Latency into the Platform and Increase the Number of Times the Mice across the Target Area in MWM Test

**Figure 2:**
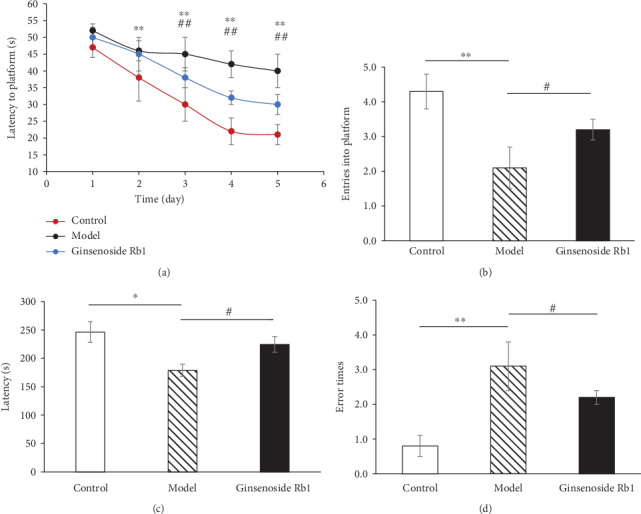
Ginsenoside Rb1 improved memory and cognition of STZ-lesioned mice in behavioral tests (*n* = 8-10). Data are expressed as mean ± SEM. (a) Ginsenoside Rb1 shortened the latency into the platform and increased entry times across the target area of mice in the MWM test. (b) Ginsenoside Rb1 increased entry times across the target area of mice in the MWM test. (c) Ginsenoside Rb1 prolonged the latency time of mice in the step-down test. (d) Ginsenoside Rb1 decreased errors of mice in the step-down test. ^∗^*p* < 0.01 and ^∗∗^*p* < 0.01, the model group vs. the control group; ^##^*p* < 0.01 and ^#^*p* < 0.05, the ginsenoside Rb1 group vs. the model group.

On the first day of navigation trial, there was no significant difference on the latency of mice among all groups. As training progressed, the latency of all groups decreased at different rates. From the second day, the model group showed the longest searching time (*p* < 0.01 vs. the control group, Figure 2(a)). During the last three days, the mice of the ginsenoside Rb1 group exhibited less searching time than the model mice (*p* < 0.01 vs. the model mice).

Probe test was conducted 24 hours after the navigation trial. The number of times the mice reached the platform was markedly reduced in the model mice (*p* < 0.01 vs. the control mice, Figure 2(b)), and ginsenoside Rb1 significantly increased that number (*p* < 0.05 vs. the model mice, Figure 2(b)).

#### 3.2.2. Ginsenoside Rb1 Prolonged Latency Time and Decreased Errors of Mice in Step-Down Test

Latency and errors are the two indices in the step-down test [25]. Latency represents the time staying at the elevated platform, and the number of errors represents the number of times the mice jumped off the platform. In our results, the model mice showed shorter escape latencies and more errors compared with the control mice (*p* < 0.05 or *p* < 0.01 vs. the control mice, Figures 2(c) and 2(d)).

In the ginsenoside Rb1 group, the latencies were increased, and errors were reduced. In other words, ginsenoside Rb1 markedly blocked the memory dysfunction of the model mice in the step-down test (*p* < 0.05 vs. the model mice, Figures 2(c) and 2(d)).

### 3.3. Ginsenoside Rb1 Improved Glucose Intolerance and Insulin Resistance Induced by STZ

**Figure 3:**
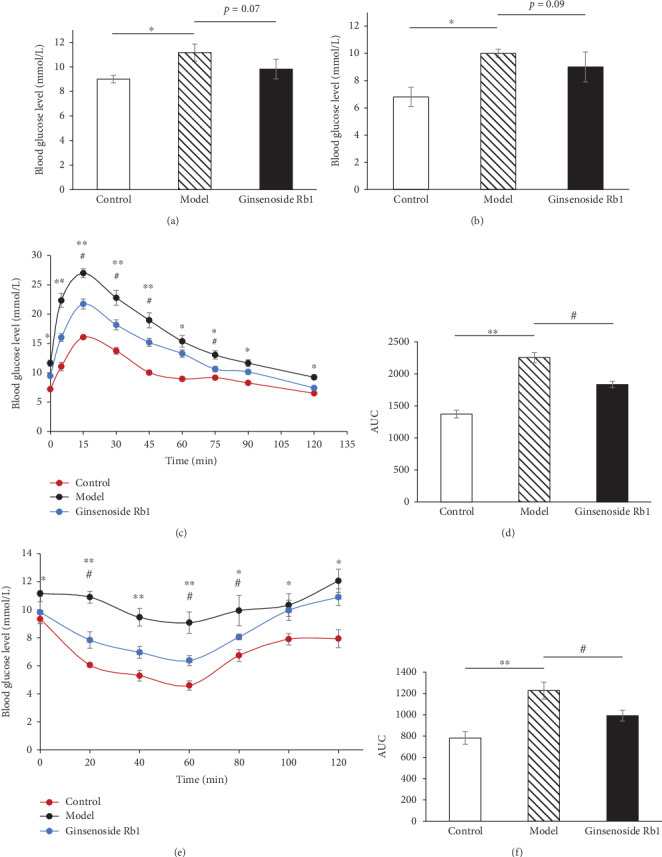
Ginsenoside Rb1 improved glucose intolerance and insulin intolerance induced by STZ (*n* = 8-10). Data are expressed as mean ± SEM. (a, b) Ginsenoside Rb1 decreased basal glucose levels of mice under both feeding and fasting conditions. (c, d) Ginsenoside Rb1 improved glucose intolerance induced by STZ. (e, f) Ginsenoside Rb1 improved insulin resistance induced by STZ. ^∗^*p* < 0.05, ^∗∗^*p* < 0.01, the model group vs. the control group; ^#^*p* < 0.05, the ginsenoside Rb1 group vs. the model group.

In the model group, the basal glucose levels of mice under both feeding and fasting conditions were significantly increased (*p* < 0.05 vs. the control group, Figures 3(a) and 3(b)). While ginsenoside Rb1 could decrease the elevated glucose level to some extent, our results showed that there is no significant decrease (*p* = 0.07 or 0.09 vs. the model group, Figures 3(a) and 3(b)). In the GTT experiment, compared with the control group, mice with STZ injection showed a significant impairment in glucose metabolism (*p* < 0.05 or *p* < 0.01 vs. the control group, Figures 3(c) and 3(d)). Ginsenoside Rb1 markedly improved glucose tolerance when compared to the model mice (*p* < 0.05 vs. the model group, Figures 3(c) and 3(d)). In the ITT experiment, STZ consistently caused severe insulin resistance in the model group (*p* < 0.05 or *p* < 0.01 vs. the control group, Figures 3(e) and 3(f)), which could be largely relieved by ginsenoside Rb1 treatment (*p* < 0.05 vs. the model mice. Figures 3(e) and 3(f)).

### 3.4. Ginsenoside Rb1 Inhibited the Activity of Cdk5/p35 *In Vivo* and Upregulated the Expression of NMDAR1 and IDE in the Hippocampus of STZ-Lesioned Mice

#### 3.4.1. Ginsenoside Rb1 Suppressed Cdk5/p35 Activity *In Vivo*

**Figure 4:**
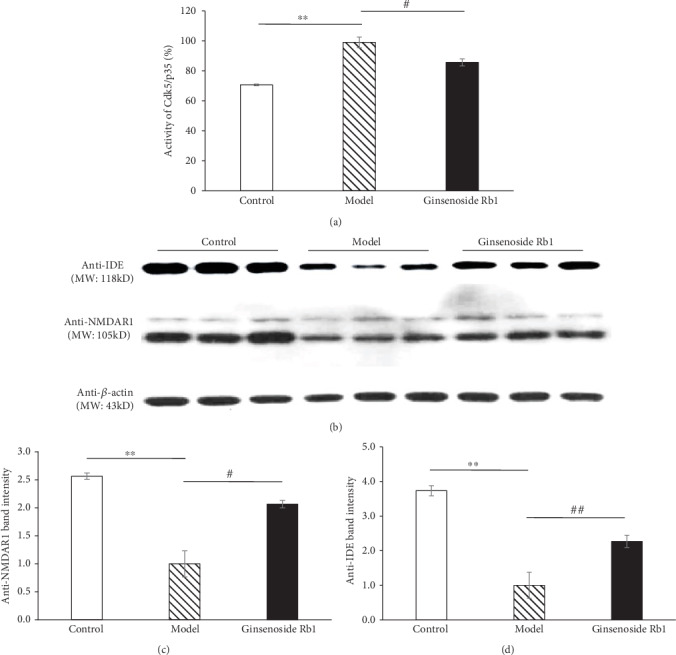
Ginsenoside Rb1 inhibited the activity of Cdk5/p35 *in vivo* and increased the expression of NMDAR1 and IDE in the hippocampus (*n* = 8-10). Data are expressed as *mean* ± *SEM*. (a) Ginsenoside Rb1 suppressed Cdk5/p35 activity *in vivo*. (b–d) Ginsenoside Rb1 unregulated NMDAR1 and IDE expression in the hippocampus of STZ-induced mice. ^∗∗^*p* < 0.01, the model group vs. the control group; ^#^*p* < 0.05 and ^##^*p* < 0.01, the ginsenoside Rb1 group vs. the model group.

In the model group, the activity of Cdk5/p35 in the mouse brain was increased significantly (*p* < 0.01 vs. the control mice, Figure 4(a)). Ginsenoside Rb1 showed a remarkable suppression on Cdk5/p35 activity (*p* < 0.05 vs. the model mice, Figure 4(a)).

#### 3.4.2. Ginsenoside Rb1 Upregulated NMDAR1 and IDE Expression in the Hippocampus

Compared with the control group, the expression of NMDAR1 and IDE was significantly reduced in the hippocampus of the model mice (*p* < 0.01 vs. the control group, Figures 4(b)–4(d)). Compared with the model mice, the ginsenoside Rb1 group exhibited a significant increase in NMDAR1 and IDE expression in the hippocampus (*p* < 0.05 and *p* < 0.01 vs. the model group, Figures 4(b)–4(d)).

## 4. Discussion

Increasing evidence indicates that an increased risk for AD is highly relevant with insulin resistance [26]. Ginsenoside Rb1 shows positive effects in treating both AD and DM in a clinical setting. In our study, using the STZ-induced mouse model confirmed the benefit of ginsenoside Rb1 on the improvement of insulin sensitivity and cognitive function.

In the present study, we conducted two behavioral tests, including the MWM test and step-down test, to demonstrate that ginsenoside Rb1 significantly reduced the impairment of learning and memory in the STZ-induced mouse model. In addition, ginsenoside Rb1 improved glucose tolerance of mice by increasing insulin sensitivity. To elucidate the mechanisms of its beneficial effects, further research was carried out.

It was reported that high-glucose exposure significantly upregulated the expression of Cdk5/p35 in hippocampal neurons with a concomitant increase in the Cdk5/p35 activity [27]. In addition, researchers found that there was a striking increase of Cdk5/p35 activity in human AD brains compared with non-AD brains [28–30]. These results show that Cdk5/p35 may be a bridge linking obesity with AD. It is well known that NMDAR1 plays an essential role in the learning and memory processes due to its high expression in hippocampal neurons [31]. A number of studies showed that Cdk5/p35 facilitated the degradation of NMDAR1 [32–34]. In this study, for the first time, we found that ginsenoside Rb1 could significantly inhibit the activity of Cdk5/p35 both *in vitro* and *in vivo*. Also, it was discovered that ginsenoside Rb1 increased the NMDAR1 expression in the hippocampus.

In the brains of aging and AD patients, a remarkable decrease of IDE levels was noted [35]. A close correlation between mutations of the IDE gene and AD risks has been reported in many genetic studies [36–37]. In addition, Miller et al. found that high A*β* peptide levels in the brain are always complemented with low IDE activity *in vivo*, which suggested that the regulation of the IDE activity *in vivo* may reduce the risk for AD to some extent [38]. Lastly, increased levels of A*β* were detected in the brains of IDE knockout mice [39], and decreased levels were detected in the brain of mice with an overexpression of IDE [40]. Consistent with the above findings, we found that STZ-lesioned mice showed a remarkable decline in memory and cognition, accompanied with markedly reduced IDE expression in the hippocampus. Ginsenoside Rb1 further reduced the impairment of learning and memory of STZ-lesioned mice and increased the IDE expression in the hippocampus.

Hyperglycaemia induced by STZ leads to progressive insulin resistance of the peripheral tissues, which results in mice being unresponsive to insulin [41]. Also, it has been reported that diabetes induced by STZ decreases IDE levels in the brains of rats [42]. Villa-Pérez et.al reported that liver-specific ablation of IDE leads to hepatic insulin resistance and glucose intolerance without affecting insulin clearance in mice [43]. Consistent with their results, STZ-lesioned mice showed severe insulin resistance, accompanied with significantly reduced IDE expression in the hippocampus in the present study. Ginsenoside Rb1 improved glucose intolerance and insulin resistance induced by STZ through upregulating the IDE expression in the brain.

In conclusion, we provide evidence that ginsenoside Rb1 reduced memory impairment and improved glucose intolerance of mice induced by STZ. These beneficial effects of ginsenoside Rb1 is most likely mediated by upregulating the expression of NMDAR1 and IDE in the hippocampus through inhibiting the activity of Cdk5/p35. This work will be of great importance in illustrating the mechanisms of ginsenoside Rb1 for improving cognitive ability and better understanding the relationship between diabetes and AD.

## Data Availability

All data used during the study are available in the article and can be solicited from the corresponding author.

## Abbreviations

AD: Alzheimer's disease
DM: Diabetes mellitus
IGF: Insulin growth factor
A*β*: Amyloid beta
GSK3*β*: Glycogen synthase kinase 3*β*
T1DM: Type 1 diabetes
NMDAR1: N-methyl-daspartate receptor type 1
MWM: Morris water maze
IDE: Insulin-degrading enzyme
Cdk5: Cyclin-dependent kinase 5.
